# Genu Recurvatum after Tibial Tuberosity Fracture

**DOI:** 10.1155/2013/952978

**Published:** 2013-04-22

**Authors:** Senthil T. Nathan, Shital N. Parikh

**Affiliations:** Division of Orthopaedic Surgery, Cincinnati Children's Hospital Medical Center, Cincinnati, OH 45229, USA

## Abstract

Fractures of the tibial tuberosity are infrequent injuries that occur during adolescence. Displaced tibial tuberosity fractures are typically treated with open reduction and internal fixation. Since these fractures occur at or near skeletal maturity, growth disturbances are not seen. This paper presents a case, the first report to our knowledge, of genu recurvatum deformity after open reduction and internal fixation of a tibial tuberosity fracture. A successful treatment plan of tibial tuberosity osteotomy with proximal tibial opening wedge osteotomy was used for the correction of genu recurvatum deformity and to maintain appropriate patellar height. At eighteen-month followup, the deformity remains corrected with satisfactory functional results. This case highlights the importance of recognition of potential complications of fracture management in adolescence.

## 1. Introduction

In 1955, Blount [1] first mentioned the theoretical complication of growth arrest leading to genu recurvatum after tibial tuberosity fracture. Since then, several authors have reported excellent clinical outcomes after the treatment of these fractures [2–9]. Though these studies have raised concern about the potential for growth arrest and genu recurvatum as a complication of treatment of these fractures, no such clinical case has been reported in the English literature. The main reason for the absence of this complication is that these fractures typically occur near skeletal maturity, when relatively little, if any, skeletal growth is remaining [5]. However, with increasing sports participation at a younger age and increasing competitiveness, several injuries once thought to be rare in the skeletally immature patients have been increasingly recognized [10]. This case report is on a skeletally immature patient who developed genu recurvatum deformity following the treatment of a tibial tuberosity fracture, which required a subsequent corrective osteotomy. To our knowledge, this is the first report of genu recurvatum deformity following the management of a tibial tuberosity fracture. Recognition of this complication may help to raise awareness about the possibility of such a complication and thus potentially avoid, anticipate, or manage them in the future. The patient was informed that the data regarding his condition would be submitted for publication and consent was obtained.

## 2. Case Report

**Figure 1:**
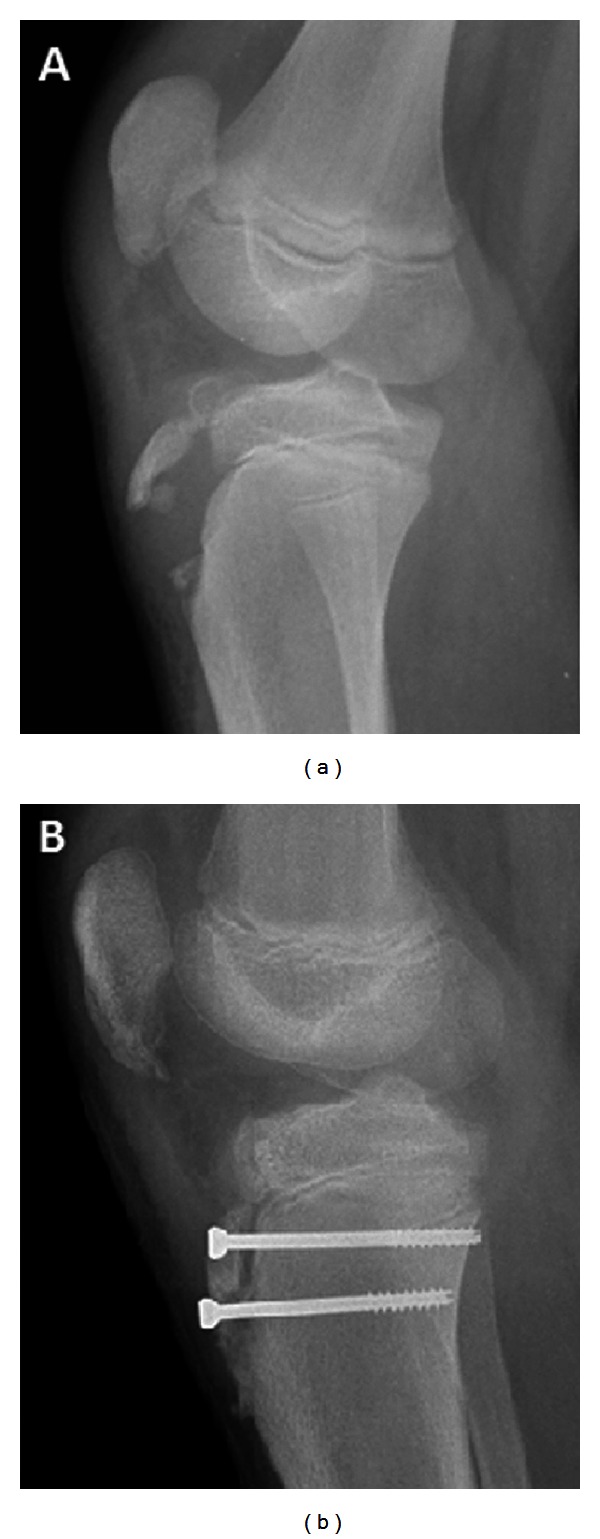
Initial injury radiographs show (a) a displaced comminuted fracture of the tibial tuberosity. (b) It was stabilized by open reduction and internal fixation using two 4.0 mm partially threaded bicortical cancellous screws.

An 11-year-old African American boy presented to the emergency department at our institution for an injury to his left knee which he sustained during basketball. He sustained the injury when trying to take off to shoot the basketball. Physical examination and radiographic evaluation confirmed a displaced tibial tuberosity fracture (Figure 1(a)). Based on Ogden classification, the fracture pattern was Type III B [5].

The patient was taken to the operating room within 12 hours of his presentation, and open reduction and internal fixation of the tibial tuberosity fracture were performed using two 4.0 mm cannulated screws (Figure 1(b)). Postoperatively, an above-knee cast was applied for 4 weeks. Following cast removal, he started physical therapy for about 6 weeks for range of motion exercises and strengthening of his leg. The fracture healed uneventfully, and 6 months later, he was discharged to full activities without restrictions, with advice to follow up if needed. 

**Figure 2:**
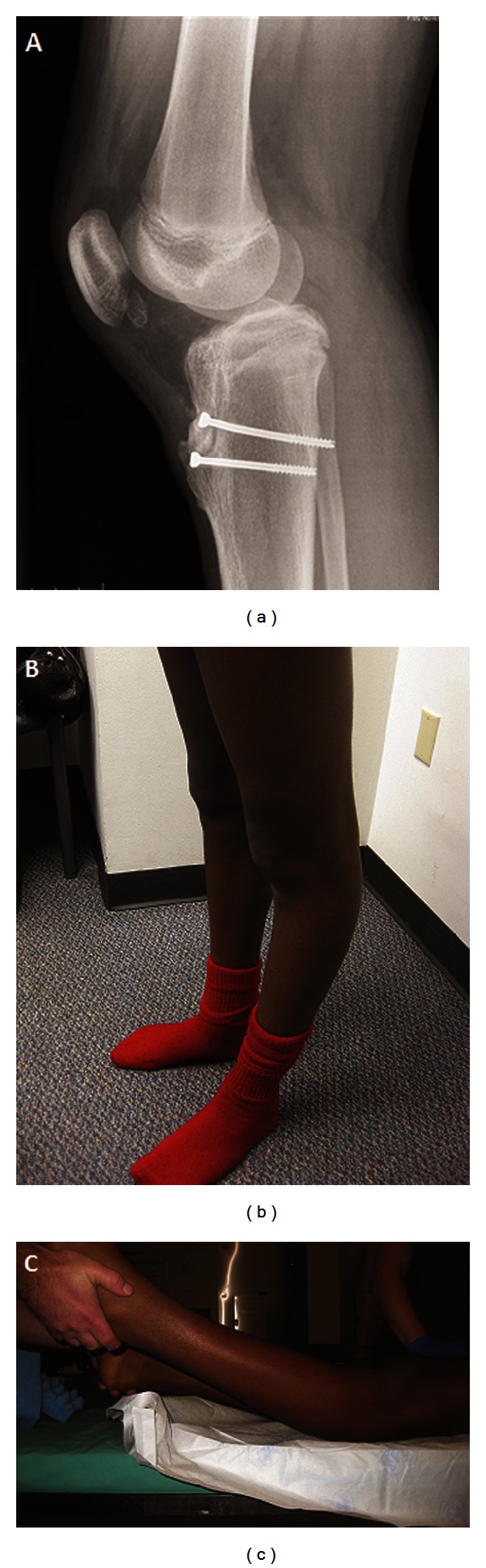
Three years after internal fixation, a genu recurvatum deformity is seen on (a) radiograph and (b, c) clinically. The radiograph shows loss of normal posterior slope of tibia and anterior subluxation of femur.

**Figure 3:**
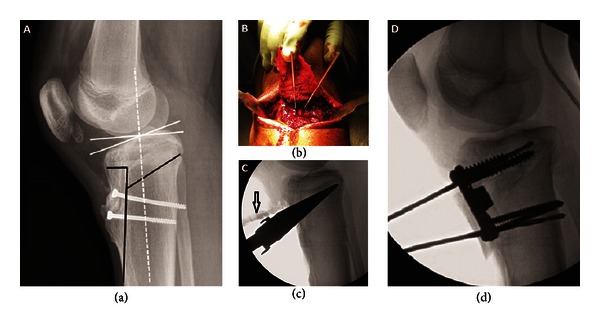
(a) Preoperative planning shows a 13° anterior slope (white lines). The planned osteotomy sites are marked (black lines). (b) At surgery, the tibial tuberosity osteotomy was hinged proximally, and two K-wires were placed to mark the plane of opening wedge osteotomy. (c) Fluoroscopic images show the 15 mm wedge used to open the osteotomy site. The arrow shows the tibial tuberosity osteotomy. (d) Tentative stabilization of tibial tuberosity fragmented using two 3.2 mm drill bits in order to check for patellar height and tracking prior to definitive fixation.

Three years later, he presented to our clinic with a progressive deformity and anterior knee pain, which was limiting his ability to play football, basketball, and skateboarding. Physical examination in standing and supine position revealed 30 degrees of hyperextension of the left knee compared to 5 degrees of hyperextension of the knee on the right side (Figure 2). He had tenderness to palpation around the tibial tuberosity. Evaluation of the lateral radiograph revealed closure of the anterior aspect of proximal tibial physis and tibial tuberosity physis with hardware in situ. The proximal tibial geometry was altered with an anterior tibial slope of 13 degrees and the absence of the normal tibial tuberosity prominence (Figure 3(a)). Magnetic resonance imaging of the knee showed no evidence of chondral, ligamentous, or degenerative changes. The patient was recommended surgical intervention to correct his deformity.

**Figure 4:**
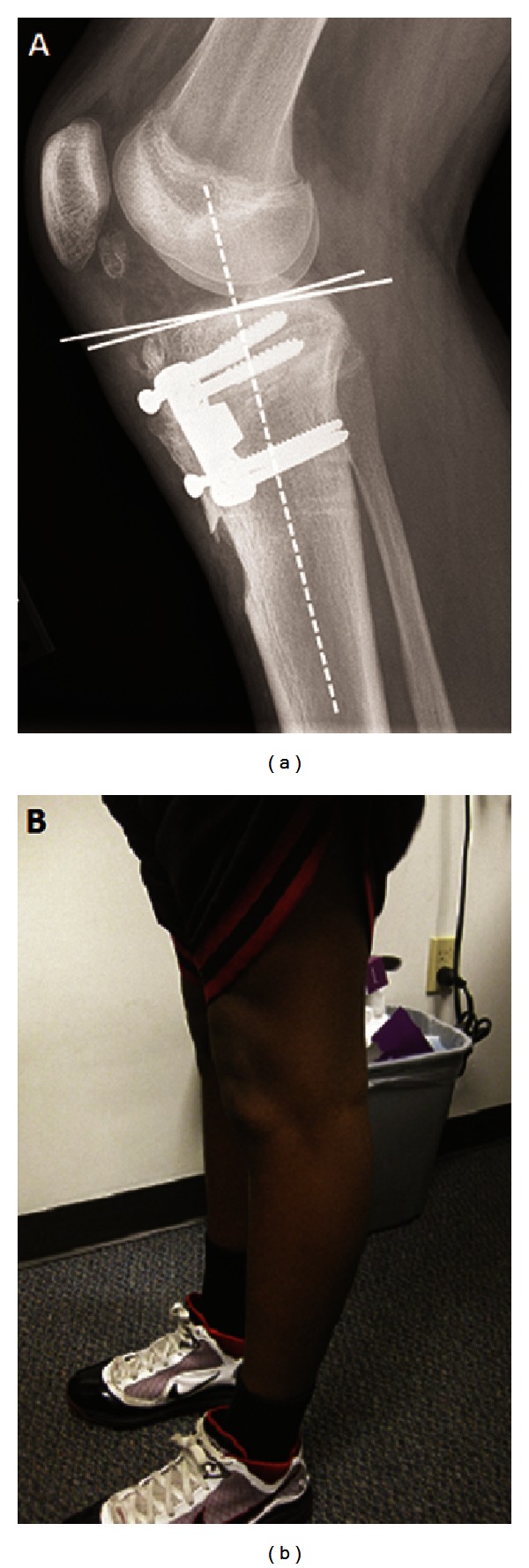
(a) Follow-up radiograph at 18 months shows a wellhealed osteotomy with 4° posterior slope (white lines) and (b) clinical correction of genu recurvatum deformity.

At surgery, the tibial tuberosity and proximal tibial shaft were exposed using previous skin incision. The screws from the previous surgery were removed with assistance of fluoroscopy and debridement of the overlying bone. An extended, broad-based tibial tuberosity osteotomy was performed. The osteotomized fragment was retracted proximally. Two K-wires were inserted through the tibial tuberosity osteotomy site, directed from anterior distal to posterior proximal, starting about 3 cm below the joint line and directed obliquely to just superior to the proximal tibiofemoral joint (Figure 3(b)). The K-wires established the plane of the proximal tibial osteotomy. Using a combination of drill, oscillating saw, and osteotomes, a proximal tibial osteotomy was performed with a posterior based hinge. The osteotomy site was gradually opened to about 15 mm using tapered wedges as per the preoperative plan (Figure 3(c)). Bicortical iliac crest bone graft was harvested from ipsilateral ilium and was impacted into the opening wedge osteotomy site. A 4-hole, 15 mm trapezoidal-wedged tibial osteotomy plate (Arthrex, Naples, FL) was placed on the anteromedial aspect of the tibia and secured using two 6.5 mm proximal screws and two 4.5 mm distal screws. The osteotomized tibial tuberosity fragment with its attached patellar tendon was replaced over the proximal tibial opening wedge osteotomy site. To maintain the patellar height, the tibial tuberosity fragment was kept aligned at its proximal osteotomy cut, which moved its distal end proximally by about 15 mm. The fragment was tentatively stabilized using two 3.2 mm drill bits (Figure 3(d)). The position of the tibial tuberosity and tracking of the patella were confirmed. Definitive fixation of the tibial tuberosity fragment was then performed using a 6.5 mm cancellous screw proximal to the opening wedge osteotomy and a 4.5 mm cortical screw distal to it. A prophylactic fasciotomy was performed over the anterior compartment of the leg. Postoperatively, a knee immobilizer was used. The patient was advised nonweight bearing on his left lower extremity. Physical therapy was started 2 weeks after surgery for passive range of motion and static quadriceps exercises. His weight bearing status was advanced after the 6th postoperative week. At the latest followup, eighteen months after his second surgery, the patient had no complaints and was back to sports with no limitation of activities. Radiographic evaluation showed a posterior tibial slope of 4 degrees (Figure 4(a)) which correlated with restoration of sagittal balance on clinical examination (Figure 4(b)). There was no significant change in the patellar height between preoperative measurements (Insall-Salvati ratio: 0.79, Blackburne-Peel ratio: 0.80) and measurements at final followup (Insall-Salvati ratio: 0.81, Blackburne-Peel ratio: 0.82).

## 3. Discussion 

 Watson-Jones classified tibial tuberosity avulsion fractures into three types [5, 6]. Type I is an avulsion fracture of its distal portion, Type II is a fracture at the junction of tibial tuberosity and proximal tibial physis, and Type III is a fracture that propagates into the knee joint. Ogden modified the Watson-Jones classification and further subdivided the fracture patterns into A and B based on the degree of communition and displacement [5]. Since the tibial tuberosity physis is contiguous with the proximal tibial physis, there is an overlap between the classification systems of each. 

The tibial tuberosity develops from a secondary ossification center between the ages of 10 and 12 years, in contrast to the ossification center of proximal tibia which is present at birth [11]. As ossification of tibial tuberosity advances, it coalesces with the proximal tibial ossification center. Physiologic epiphysiodesis of the proximal tibia begins centrally in the proximal tibial epiphysis and proceeds centrifugally, with the area beneath the tuberosity fusing last. The direction of physeal closure of tibial tuberosity is from proximal to distal [5]. This pattern of physeal closure makes the tibial tuberosity more vulnerable to fracture in the adolescent, which is similar to other transitional fractures like the Tillaux fracture of the ankle. The tibial tuberosity ossification center fuses with the metaphysis by the age of 15 to 17 years [11]. Histopathologically, the transition to columnar cartilage cells at the tibial tuberosity-metaphysis junction in adolescence makes it structurally weaker than the fibrocartilage that is normally present at this junction. This transition leads to an increased incidence of avulsion fractures of the tibial tuberosity in adolescence when subjected to tensile forces [5, 6]. 

Typically, tibial tuberosity fractures occur following a sport injury requiring a takeoff or landing on one foot like basketball, football, soccer, or sprinting. The prognosis of these fractures is generally good. The theoretical complication of genu recurvatum has not been reported because most of these injuries occur when the physis is nearing normal closure. The most common ages for these fractures are between 12 and 17 years, though fractures in preadolescents (8–12 years) have been reported [5, 7–9]. At a younger age, growth arrest of the tibial tuberosity physis due to any cause would lead to asymmetric growth of the proximal tibia, genu recurvatum deformity, or a decrease in the normal 10° posterior tibial slope. The anterior tibial slope of 13° in our patient would equate to a total of 23° loss of tibial slope. Since our patient had 25° increased hyperextension of the involved knee compared to the contralateral side, most of his deformity would be considered osseous. It is important to distinguish osseous deformities from soft-tissue deformities due to capsuloligamentous incompetence because the former has better results after surgical correction [12]. The sagittal plane deformity after tibial tuberosity growth arrest (genu recurvatum) is different from the typical coronal plane deformities after proximal tibial physeal growth disturbances (genu valgum, genu varum).

Similar to other physeal injuries in the body, the etiology of growth arrest in this case could be either the injury or its treatment. Genu recurvatum in children and adolescence has been reported in the literature following skeletal traction with proximal tibial pin or distal femoral pin, long-term immobilization, ipsilateral lower limb fractures, Osgood-Schlatter's disease, osteomyelitis, and tibial tuberosity osteotomy [7, 12–14]. The recognition of preventable causes of genu recurvatum, including proximal tibial traction pin and tibial tuberosity osteotomy, is important and has led to an almost complete abandonment of these procedures in the skeletally immature. The management of tibial tuberosity fractures in the younger patient should follow the principles of management of physeal fractures, including cautious approach during fixation across the physis, removal of hardware after fracture healing, and adequate followup until skeletal maturity. 

Several techniques for correction of genu recurvatum have been reported in the literature, including opening wedge osteotomy above the tibial tuberosity [12], opening wedge osteotomy below tibial tuberosity [12, 13], closing wedge osteotomy [15], gradual correction using external fixator [16, 17], and opening wedge osteotomy with tibial tuberosity osteotomy [12]. Besides correction of the deformity, an opening wedge osteotomy has the advantage of regaining the anterior length lost due to growth arrest. Though an opening wedge osteotomy distal to tibial tuberosity can correct genu recurvatum, it could lead to anterior prominence of the tibia and its effect on correction of tibial slope would be less than an osteotomy performed closer to the joint surface [12]. This follows the principles of deformity correction that maximum correction would be achieved at the site of deformity rather than away from it. An opening wedge osteotomy proximal to the tibial tuberosity can lead to a decrease in patellar height [12, 18]. Moroni et al. [12] reported satisfactory results of opening wedge osteotomy with tibial tuberosity osteotomy when compared to closing wedge osteotomy or opening wedge osteotomy without tibial tuberosity osteotomy. The advantages of an opening wedge osteotomy proximal to tibial tuberosity combined with tibial tuberosity osteotomy, as in our case, are greater ability to adjust patellar height, greater correction of deformity, more simplistic technique, and no need for concomitant fibular osteotomy since tibial osteotomy is proximal to the proximal tibiofibular joint. 

## 4. Conclusion

This case of an 11-year-old boy with genu recurvatum after open reduction and internal fixation treatment of tibial tuberosity fracture highlights the importance of recognition and management of potential complications of fracture management in adolescence.
